# Fatigue Survival and Biomechanical Behavior of Two-piece Titanium and Zirconia Dental Implants

**DOI:** 10.4317/jced.64022

**Published:** 2026-04-25

**Authors:** Jefferson David Melo de Matos, Guilherme da Rocha Scalzer Lopes, Nathália de Carvalho Ramos, Mário Alexandre Coelho Sinhoreti, Alexandre Luiz Souto Borges, Daher Antonio Queiroz, Marco Antonio Bottino

**Affiliations:** 1 PhD, Department of Biomaterials, Dental Materials and Prosthodontics, São Paulo State University (Unesp), Institute of Science and Technology, São José dos Campos, Sao Paulo, Brazil; 2 Assistant Professor, Dental Materials and Prosthodontics, São Paulo State University (Unesp), Institute of Science and Technology, São José dos Campos, Sao Paulo, Brazil; 3 Full professor, Department of Restorative Dentistry, Dental Materials Division, Piracicaba Dental School (FOP UNICAMP), Piracicaba, Sao Paulo, Brazil; 4 Associate Professor, Dental Materials and Prosthodontics, São Paulo State University (Unesp), Institute of Science and Technology, São José dos Campos, Sao Paulo, Brazil; 5 DDS, MSc, PhD Associate Professor, Department of Restorative Sciences and Public Health Dentistry, Nova Southeastern University College of Dental Medicine, Fort Lauderdale, Florida, USA; 6 Adjunct Associate Professor, Department of Restorative Dentistry &amp; Prosthodontics, The University of Texas Health Science Center at Houston (UTHealth) School of Dentistry, Houston, Texas, USA; 7 Full professor, Department of Biomaterials, Dental Materials and Prosthodontics, São Paulo State University (Unesp), Institute of Science and Technology, São José dos Campos, Sao Paulo, Brazil

## Abstract

**Background:**

Titanium implants are considered the gold standard for oral rehabilitation; however, zirconia implants have emerged as a metal-free alternative with promising esthetic and biological properties. Despite these advantages, limited evidence is available regarding their long-term fatigue behavior and fracture resistance.

**Material and Methods:**

In this in vitro experimental study conducted at São Paulo State University (UNESP), Brazil, forty implants were divided into two groups (n = 20): zirconia implants (ZZ) and titanium implants (TZ) (4.1 × 10 mm). Implants were embedded in polyurethane blocks according to ISO 14801:2016 specifications. Monolithic zirconia crowns were fabricated using CAD/CAM technology and cemented onto zirconia abutments. All assemblies underwent thermomechanical aging (2×106 cycles, 200 N, 2 Hz). Fatigue resistance was assessed using a stepwise loading protocol. Crown-abutment misfit was measured microscopically, and torque loss after fatigue was recorded. Statistical analysis was performed using Mann-Whitney U tests and Kaplan-Meier survival analysis ( = 0.05).

**Results:**

Zirconia implants showed significantly greater fracture resistance (1123.73 ± 153.61 N) than titanium implants (818.67 ± 42.41 N) (p = 0.037). No significant difference in fatigue survival was observed between groups (p = 0.097). The zirconia group presented greater crown-abutment misfit (39.63 ± 10.1 m) than the titanium group (25.09 ± 4.6 m) (p &lt; 0.001). Catastrophic implant body fractures were more frequent in the zirconia group, whereas titanium implants exhibited more repairable failure modes.

**Conclusions:**

Zirconia implants demonstrated similar fracture resistance but showed catastrophic implant fractures, whereas titanium implants presented repairable failure patterns.

## Introduction

Dental implants are a predictable and widely used treatment modality for the rehabilitation of partially or completely edentulous patients (1 - 4). Since the osseointegration protocol proposed by Brånemark, titanium implants have been considered the gold standard due to their excellent biocompatibility, mechanical stability, and long-term clinical success (5 - 9). Continuous advances in implant surface treatments, macrogeometry, and prosthetic connections have further improved biological integration and mechanical reliability, contributing to the widespread clinical use of titanium-based implant systems (9). Despite their high clinical success, increasing esthetic demands and the growing preference for metal-free restorations have stimulated the development of ceramic implant systems (9 - 11). Among these materials, zirconia has attracted considerable attention due to its favorable biological and esthetic characteristics. Zirconia is a polycrystalline ceramic biomaterial characterized by high biocompatibility, reduced bacterial adhesion, and favorable soft-tissue response (1 - 5). Additionally, its tooth-like color minimizes the risk of gray discoloration of peri-implant tissues, particularly in patients with thin gingival biotypes or in anterior esthetic regions (12 - 16). However, zirconia exhibits mechanical properties that differ from those of titanium. Although it presents high compressive strength and stiffness, zirconia behaves as a brittle material and may be more susceptible to fracture and fatigue under cyclic loading conditions (12 - 16). Furthermore, factors such as manufacturing defects, low-temperature degradation, and microstructural flaws may influence its long-term mechanical reliabilityn(17 - 19). To overcome these limitations, hybrid configurations combining zirconia and titanium components have been proposed to improve fracture resistance. Nevertheless, the incorporation of metallic components may introduce additional interfaces susceptible to mechanical complications over time (12 - 16 , 18 - 20). Another limitation is that most zirconia implants currently available are designed as one-piece systems, which restrict prosthetic flexibility and limit restorative options (18 - 20). The development of two-piece zirconia implant systems has been proposed to overcome these limitations by allowing screw-retained restorations and improved prosthetic versatility while maintaining the esthetic and biological advantages of ceramic materials (4 , 10 , 18 - 20). Therefore, the aim of this in vitro study was to evaluate the biomechanical behavior and fatigue performance of two-piece zirconia and titanium implant systems restored with zirconia abutments and monolithic zirconia crowns. The null hypothesis was that both implant systems would demonstrate similar biomechanical behavior under simulated functional loading.

## Material and Methods

This in vitro experimental study was conducted at the Biomaterials and Biomechanics Research Laboratory, São Paulo State University (UNESP), Brazil, between March and August 2024. The study followed ISO 14801:2016 guidelines for dynamic fatigue testing of dental implants. As this study was conducted entirely in vitro and did not involve human or animal subjects, institutional ethical approval was not required. Sample size calculation was based on previously published biomechanical investigations (28 , 29). Considering the power of 80% and a significance level of 5%, a minimum sample size of 18 specimens per group was estimated. Therefore, 20 implants were included in each group to compensate for potential experimental losses. 1. Specimen Preparation Forty implants were divided into two groups (n = 20): zirconia implants (Straumann PURE Ceramic Implant System, Institut Straumann AG, Basel, Switzerland) and titanium implants (Straumann Bone Level Implant System, Institut Straumann AG, Basel, Switzerland), both with dimensions of 4.1 mm in diameter and 10 mm in length and a regular platform configuration (RP). All implants featured an internal connection design and were restored using manufacturer-recommended abutments. Cylindrical polyurethane resin blocks were fabricated to simulate cancellous bone in accordance with ISO 14801:2016 specifications. The blocks exhibited an elastic modulus of 3.6 GPa (34) and were produced using a vacuum pressurization technique to minimize air entrapment. Implants were installed following the manufacturer's drilling protocol and torqued to 35 Ncm. A vertical exposure of 3 mm above the resin block was maintained to simulate crestal bone resorption, as recommended by ISO 14801 (35). Prosthetic abutments (Variobase RB/WB 5.5 mm and PUREbase CI/RD 5.5 mm; Straumann Dental Implant System, Basel, Switzerland) were connected using a calibrated torque wrench at 35 Ncm, according to the manufacturer's recommendations. 2. Crown Fabrication and Cementation Monolithic zirconia crowns were fabricated from 3Y-TZP zirconia blocks (IPS e.max ZirCAD, Ivoclar Vivadent, Schaan, Liechtenstein), milled using a CAD/CAM system and sintered according to manufacturer instructions. For cementation, a dual-cure resin cement (Variolink Esthetic DC, Ivoclar Vivadent) was used in combination with an MDP-containing ceramic primer (Monobond Plus, Ivoclar Vivadent). Surface treatment included airborne-particle abrasion with 50 m Al2O3. Monolithic zirconia crowns reproducing a maxillary lateral incisor were fabricated using a CAD/CAM system. Crowns were milled from pre-sintered monolithic zirconia blocks and sintered according to the manufacturer's recommendations. Prior to cementation, abutments were airborneparticle abraded with 50 m aluminum oxide and treated with a ceramic primer containing MDP. Crowns were cemented to their respective bases using dualcure resin cement under standardized seating pressure. Excess cement was removed and polymerization was completed using an LED curing unit. 3. Thermomechanical Aging Thermomechanical aging was performed using a chewing simulator (CS-4.8, SD Mechatronik, Feldkirchen-Westerham, Germany), previously validated for simulating long-term oral conditions (42). All specimens were subjected to 2 × 106 loading cycles at 200 N and 2 Hz in distilled water at 37 °C to simulate long-term clinical function (28 , 29). Subsequently, fatigue testing was conducted using a universal testing machine (Instron 8872, Instron Corp., Norwood, MA, USA), calibrated in accordance with ISO 14801:2016 standards (35). 4. Fatigue Testing Following aging, fatigue resistance was evaluated using a stepwise loading protocol in a mechanical cycling device. Specimens were loaded at a 30° angle relative to the implant long axis (31 , 32). The initial load was set at 100 N and progressively increased at defined intervals until failure occurred. Failure modes were classified as crown fracture, abutment fracture, or implant body fracture (33). 5. Misfit Analysis Crown-abutment misfit was evaluated using a digital microscope (VHX-7000, Keyence Corp., Osaka, Japan) and scanning electron microscopy (SEM; JSM-6610LV, JEOL Ltd., Tokyo, Japan), following established protocols for marginal adaptation assessment (30). Measurements were obtained at four locations (buccal, palatal, mesial, and distal). To ensure reliability, 20% of the samples (8 implants, 32 measurement sites) were re-evaluated, and intra- and inter-examiner agreement was assessed using the Kappa test (43). 6. Statistical Analysis All statistical analyses were performed using SPSS software (IBM SPSS Statistics for Windows, version 25.0; IBM Corp., Armonk, NY, USA). Data normality was assessed using the Shapiro-Wilk test. Between-group comparisons were conducted using the Mann-Whitney U test, while fatigue survival was analyzed using Kaplan-Meier curves and the log-rank test. The significance level was set at = 0.05.

## Results

1. Maximum Fracture Load Zirconia implants demonstrated significantly higher fracture resistance (1123.73 ± 153.61 N) than titanium implants (818.67 ± 42.41 N). This difference was statistically significant (p = 0.037) (Table 1).

Table 1

3.2. Torque Loss Both implant groups exhibited torque loss after thermomechanical aging. The titanium group showed a greater reduction in torque compared with the zirconia group (Table 2).

Table 2

**Figure 1:**
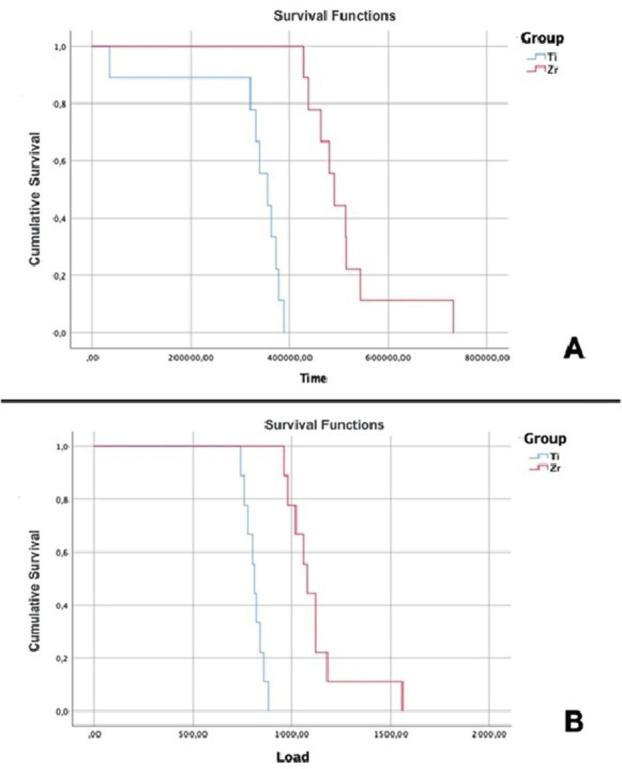
Survival graph of groups. Figure 1A: Survival graph of groups as a function of time (cycles). Figure 1B: Survival graph of groups as a function of load (cycles).

3.3 Fatigue Survival Analysis No specimen failed during the initial 2×106 cycles at 200 N. Subsequent stepwise loading revealed no statistically significant difference in fatigue survival between groups (p = 0.097) (Fig. 1, Tables 3,4).

1

Table 3

Table 4

**Figure 2:**
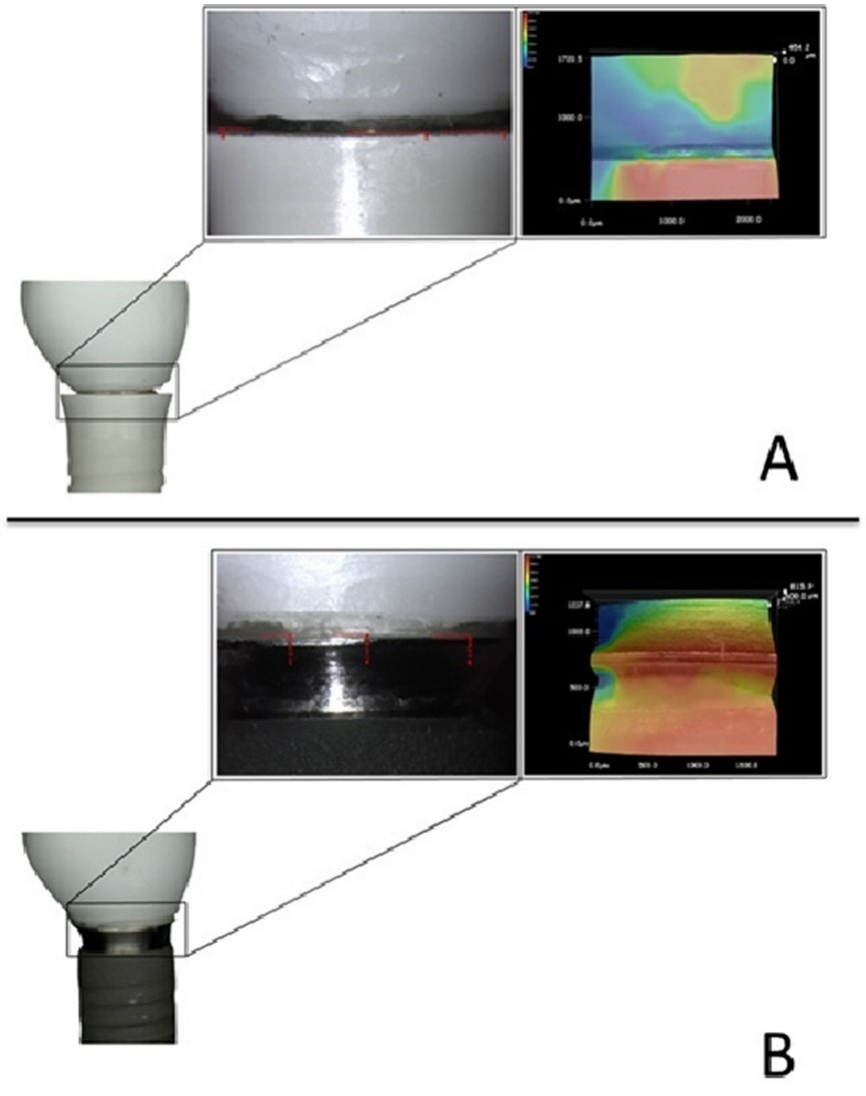
Crown–abutment misfit and experimental configuration of the evaluated implant systems. (A) Zirconia implant system (ZZ) restored with zirconia abutment and monolithic zirconia crown; (B) Titanium implant system (TZ) restored with zirconia abutment and monolithic zirconia crown. Representative 2D (×477) and 3D images obtained by digital microscopy illustrate the crown–abutment interface and highlight the regions of greatest misfit, while also providing a visual representation of the materials and configurations used in each experimental group.

3.4 Crown-Abutment Misfit The zirconia group presented significantly greater misfit values (39.63 ± 10.1 m) compared with the titanium group (25.09 ± 4.6 m) (p &lt; 0.001) (Fig. 2, Table 5).

2

Table 5

Failure modes differed between groups: zirconia implants exhibited catastrophic implant body fractures, whereas titanium implants mainly demonstrated repairable prosthetic failures. A detailed failure mode analysis was performed to characterize the mechanical behavior of the implant systems after fatigue testing. Failures were classified into three categories: 1) crown fracture, 2) abutment fracture, and 3) implant body fracture. The zirconia implant group predominantly exhibited catastrophic implant body fractures, characterized by complete structural failure with no possibility of clinical repair. These fractures typically originated at the cervical region and propagated through the implant body, consistent with tensile stress concentration patterns. In contrast, the titanium implant group mainly showed prosthetic-related failures, including abutment deformation and screw-related complications, which are considered clinically manageable. No isolated crown fractures were observed in either group, indicating adequate bonding performance of the monolithic zirconia restorations. Fractographic inspection (qualitatively assessed during microscopy analysis) suggested crack initiation at regions of stress concentration, followed by rapid crack propagation in zirconia specimens, whereas titanium specimens exhibited signs of plastic deformation prior to failure. This reinforces the distinct failure mechanisms associated with brittle versus ductile materials.

## Discussion

The present study investigated the fatigue survival and biomechanical behavior of two-piece zirconia and titanium implant systems restored with zirconia abutments and monolithic zirconia crowns. The null hypothesis was rejected, as significant differences were observed between the materials. Zirconia implants demonstrated higher fracture resistance than titanium implants; however, failures were predominantly catastrophic, involving fracture of the implant body. In contrast, titanium implants exhibited lower fracture strength but more favorable failure patterns that were mainly restricted to prosthetic components (6 , 12 , 15). The distinct performance of the implant systems can be explained by the intrinsic mechanical properties of titanium and zirconia. Titanium exhibits high fracture toughness and ductility, allowing plastic deformation and effective dissipation of mechanical energy under cyclic loading. Conversely, zirconia behaves as a brittle ceramic with high compressive strength but limited resistance to crack initiation and propagation (2 , 8 , 25). Under fatigue conditions, stress concentrations play a critical role in determining failure behavior. These stresses are not uniformly distributed along the implant-abutment-crown complex but tend to localize in critical regions, particularly at the cervical portion of the implant, the implant-abutment interface, and areas with geometric discontinuities (15 , 33 , 44). In this context, previous biomechanical studies have demonstrated that zirconia components are more susceptible to failure due to localized stress peaks. The study published by Datte et al. 2019 (44) paper zirconia abutment failure reported that zirconia abutments fail more frequently than titanium ones, primarily due to higher stress concentrations at the connection interface. These findings are in agreement with the present results, in which zirconia implants exhibited catastrophic fractures, suggesting that the inability of the material to redistribute stress contributes to crack propagation once critical stress thresholds are reached. However, an important distinction should be emphasized: while the referenced study identified abutment-level failures, the present study observed failures predominantly at the implant body level. This discrepancy may be attributed to differences in implant design, connection geometry, and load transfer mechanisms (6 , 15 , 23). Stress distribution is strongly influenced by the elastic modulus mismatch between components. Zirconia has a significantly higher elastic modulus than titanium, which reduces its ability to absorb and redistribute occlusal forces. As a result, stresses are transferred more directly to the implant structure, increasing the likelihood of catastrophic failure. In contrast, titanium's lower elastic modulus allows for more favorable stress distribution, reducing peak stress concentrations and shifting failure to prosthetic components (2 , 15 , 33 , 44). The loading protocol applied eccentric forces at a 30° inclination relative to the implant axis to simulate non-axial masticatory dynamics. This configuration is known to exacerbate bending moments and amplify stress concentration at the cervical region of the implant system. Finite element and in vitro studies corroborate that oblique loading significantly increases tensile stress, particularly in ceramic systems (31 , 33 , 44). Although the fracture loads exceeded the physiological range typically reported for anterior teeth (approximately 60-170 N), this approach enables standardized and comparative evaluation of mechanical performance under extreme conditions (35 , 42). The greater crown-abutment misfit observed in the zirconia implant group may have further intensified stress concentration at the prosthetic interface. Even minimal discrepancies in marginal adaptation can act as stress risers, especially in brittle materials such as zirconia, accelerating fatigue damage accumulation (30 , 33). In contrast, the presence of a titanium inserts in the hybrid abutment configuration used with titanium implants likely contributed to improved stress distribution and mechanical stability. This supports the concept that hybrid ceramic-metal configurations can mitigate stress concentration by combining esthetic and mechanical advantages (10 , 11 , 21 , 36). The prosthetic assembly remained stable throughout thermomechanical aging, as no debonding of monolithic zirconia crowns was observed. Adhesive cementation with resin cement provided adequate retention, corroborating previous studies evaluating zirconia-based implant restorations (24 , 27 , 42). Nevertheless, the absence of debonding does not necessarily imply optimal stress distribution, as internal stresses may still accumulate within the restorative complex. These findings are consistent with previous investigations reporting distinct failure modes for zirconia and titanium implant systems. Catastrophic fractures in zirconia implants may be associated not only with stress concentration but also with microstructural defects introduced during manufacturing or sintering processes. Porosities, grain boundary flaws, and phase transformation phenomena may act as crack initiation sites under cyclic loading (8 , 25 , 36). Additionally, implant macrogeometry and surface treatment may further influence stress distribution and fatigue behavior (16 , 15). From a clinical perspective, zirconia implants have shown survival rates ranging from approximately 90.9% to 96.3%, whereas titanium implants typically demonstrate slightly higher survival rates between 95.8% and 98.6% (4 , 9 , 13 , 14). While zirconia implants offer advantages in terms of esthetics and biocompatibility, their mechanical reliability remains influenced by their brittle nature and sensitivity to stress concentration (3 , 7 , 23). Therefore, careful case selection, occlusal control, and prosthetic design are essential to minimize biomechanical complications. Despite the controlled experimental conditions and the use of standardized testing based on ISO 14801, the inherent limitations of in vitro studies must be acknowledged. Laboratory simulations cannot fully replicate the complex biomechanical environment of the oral cavity, including bone anisotropy, remodeling processes, and multidirectional loading patterns (34 , 35). Although previous studies have used finite element analysis to investigate stress distribution in implant-supported restorations (33 , 44), evidence remains limited for two-piece zirconia implant systems under fatigue conditions. Therefore, further studies combining computational modeling with long-term experimental and clinical data, preferably incorporating patient-specific parameters, are necessary to better elucidate stress distribution and optimize implant design.

## Conclusions

Within the limitations of this in vitro study, zirconia implants exhibited similar fracture resistance; however, their failure pattern was predominantly catastrophic, involving implant body fractures. In contrast, titanium implants showed lower fracture resistance but more favorable and clinically manageable failure modes, mainly restricted to prosthetic components. These findings highlight a trade-off between strength and failure behavior, emphasizing that material selection should consider not only load-bearing capacity but also the predictability and reparability of failures. Although computational studies have previously explored stress distribution in implant-supported systems, further investigations specifically focused on two-piece zirconia implants under fatigue conditions are warranted to better understand their biomechanical performance and support safer clinical application.

## Figures and Tables

**Table 1:** Maximum fracture load values for zirconia implants with zirconia abutments (ZZ) and titanium implants with zirconia abutments (TZ).

Groups	Mean ± SD	CI 95%
ZZ	1123.73 ± 153.61	(970.12 – 1277.34)
TZ	818.67 ± 42.41	(776.26 – 861.08)

CI - Confidence interval.

**Table 2:** Prosthetic screw torque and detorque (N) performance in zirconia (ZZ) and titanium (TZ) implant-abutment groups, with confidence intervals.

Groups	Mean ± SD	CI 95%
ZZ	25.2 ± 3.8a	(21.3 – 29.0)
TZ	22.8 ± 3.5b	(19.2 – 26.3)

Different letters indicate statistical difference between the groups analyzed. CI - Confidence interval.

**Table 3:** Fatigue failure values means and number of cycles to fracture of the groups analyzed.

Fatigue failure load (N)		
Groups	Mean ± SD	95% CI (Lower–Upper)
ZZ	1120 ± 59.81 a	1002.76 – 1237.23
TZ	810 ± 15.27 b	708.06 – 839.93
Number of Cycles to Fatigue Failure (N)		
Groups	Mean ± SD	95% CI (Lower–Upper)
ZZ	512,029 ± 36,385a	452,918 – 571,139
TZ	320,818 ± 30,158 b	249,503 – 392,133

Different letters indicate statistical difference between the groups analyzed. CI - Confidence interval.

**Table 4:** Estimated survival rates of the experimental groups according to the applied load and number of cycles (stepwise).

Total Cycles / Load (N)									
Group	325K / 740	335K / 760	345K / 780	355K / 800	365K / 810	375K / 820	385K / 840	395K / 860	405K / 880
TZ	88% (10)	77% (13)	66% (15)	55% (16)	44% (16)	33% (15)	22% (13)	11% (10)	0%
ZZ	100%	100%	100%	100%	100%	100%	100%	100%	100%
Total Cycles / Load (N)									
	425K / 940	435K / 960	445K / 980	465K / 1020	485K / 1060	495K / 1080	515K / 1120	545K / 1180	735K / 1560
TZ	0%	0%	0%	0%	0%	0%	0%	0%	0%
ZZ	99% (10)	88% (10)	77% (13)	66% (15)	55% (15)	44% (16)	22% (16)	11% (10)	0%

Zirconia abutments (ZZ) and titanium implants with zirconia abutments (TZ).

**Table 5:** Misfit between the crown and implant in the studied groups.

Process data	Misfit (μm)	W	p-value
ZZ	39.63 ± 10.1	135	0.001
TZ	25.09 ± 4.6	50.513	0.001

5

## Data Availability

The datasets generated during the current study are not publicly available due to ethical and confidentiality restrictions but are available from the corresponding author upon reasonable request.
